# Obesity is associated with increased risk of invasive penile cancer

**DOI:** 10.1186/s12894-016-0161-7

**Published:** 2016-07-13

**Authors:** Kerri T. Barnes, Bradley D. McDowell, Anna Button, Brian J. Smith, Charles F. Lynch, Amit Gupta

**Affiliations:** Department of Urology, University of Iowa, 200 Hawkins Dr., 3 RCP, Iowa City, IA 52242-1089 USA; Holden Comprehensive Cancer Center, University of Iowa, Iowa City, IA USA; Department of Biostatistics, University of Iowa, Iowa City, IA USA; Department of Epidemiology, University of Iowa, Iowa City, IA USA

**Keywords:** Penile cancer, Obesity, Epidemiologic study, Case control study

## Abstract

**Background:**

To validate the association between obesity and penile cancer at a population level, we conducted a matched case–control study linking the Iowa Department of Motor Vehicles Drivers’ License Database (DLD) with cancer surveillance data collected by the State Health Registry of Iowa (SHRI).

**Methods:**

All men diagnosed with invasive penile squamous cell carcinoma from 1985 to 2010 were identified by SHRI. Two hundred sixty-six cancer cases and 816 cancer-free male controls, selected from the Iowa DLD, were matched within 5-year age and calendar year strata. Body mass index (BMI) was calculated using self-reported height and weight from the DLD.

**Results:**

Conditional logistic regression was used to evaluate the association between BMI and the risk of developing invasive penile cancer. Obesity was significantly associated with an increased risk of developing penile cancer. For every five-unit increase in BMI the risk of invasive penile cancer increased by 53 % (OR 1.53, 95 % CI 1.29-1.81, *p* < 0.0001).

**Conclusion:**

We previously reported an association between obesity and higher risk of invasive penile cancer and advanced cancer stage at diagnosis in a hospital-based retrospective study. This population-based study confirms an association between obesity and invasive penile cancer.

## Background

Penile cancer accounts for 0.4–0.6 % of malignancies in the United States and Western Europe [1]. Lack of neonatal circumcision, poor genital hygiene, phimosis, human papilloma virus (HPV) infection and smoking are all risk factors for invasive penile cancer [2]. In a hospital-based retrospective study, we reported an association between obesity and higher risk of having invasive penile cancer after controlling for race and smoking status [3]. In order to validate the association between obesity and penile cancer at the population level, we conducted a matched case–control study linking the Iowa Department of Motor Vehicles Drivers’ License Database (DLD) with the State Health Registry of Iowa (SHRI), which is a member of the Surveillance, Epidemiology, and End Results (SEER) Program [4].

## Methods

Institutional Review Board approval was obtained for this study. Cancer cases were ascertained by the SHRI as part of its surveillance mandate. All male Iowa residents diagnosed at 18+ years of age between 1985 and 2010 with microscopically-confirmed invasive penile squamous cell carcinoma were identified. These criteria yielded an initial cohort of 278 cases. The DLD was obtained from the Iowa Department of Motor Vehicles. This database contains full name, date of birth, license issue date, and self-reported height and weight. Each person in the database may have multiple records that correspond to successive license renewals.

Data from the SHRI (for diagnoses made between 1985 and 2010) and the DLD (for licenses issued in 1985, 1993, 2008 and 2012) were linked using full name, social security number and date of birth. Of the 278 initially identified penile cancer cases, 266 (96 %) were successfully matched to one or more records in the DLD. A control group of males were randomly selected from the Iowa DLD, and SHRI records were checked to confirm that these men had not been diagnosed with cancer. These controls were randomly selected from within 5-year calendar year strata from 1985 to 2012 (according to time of diagnosis for cases and time of DL issuance for controls) and 5-year age strata for ages 30 through 90+. Matching was done in a 3:1 ratio, yielding 798 controls.

Body mass index (BMI) was calculated using self-reported height and weight from the DLD and categorized as at or below normal weight (<25), overweight (25–29.9), or obese (≥30) according to World Health Organization (WHO) criteria [5]. Because DL issuance dates (and corresponding BMIs) do not often coincide closely with dates of diagnoses (Table 1), linear mixed effects regression was used to estimate BMI at the time of diagnosis for each case. All available BMIs calculated from the DLD information were included in the regression analysis and modeled as a function of (1) fixed effects for linear temporal trends prior to and after diagnosis and (2) random effects for case-specific mean differences. The regression-derived estimates of BMI at diagnosis were then used in the subsequent analysis of cancer risk. This was not necessary for controls since their BMIs were calculated directly from DLs selected to coincide with the diagnosis dates of their matched cases. Conditional logistic regression was used to evaluate the association between BMI and risk of developing invasive penile cancer, while adjusting for the 5-year age and calendar year matching strata which controlled for changes in association over time. Estimated effects of BMI are reported as odds ratios, along with 95 % confidence intervals. All statistical tests were two-sided and assessed for significance at the 0.05 level.

**Table 1:** Summary of the number and timing of drivers’ licenses available for penile cancer cases relative to diagnosis

DL Issued	N	Number of licenses		Years to closest license	
		Mean (SD)	Range	Mean (SD)	Range
Before diagnosis	262	1.5 (0.7)	1–4	8.8 (6.7)	0–25
After diagnosis	90	1.3 (0.6)	1–4	6.6 (5.6)	1–22
Before or after	266	1.9 (0.9)	1–5	6.6 (5.9)	0–25

## Results

The mean age of cases and controls were 68.3 and 69.3 years, respectively. In the mixed effects regression analysis for penile cancer patients, BMIs increased at an estimated rate of 1.0 unit every 10 years (95 % CI: 0.7 - 1.3; *p* < 0.0001). A non-significant decreased rate of 0.1 units (95 % CI: -0.4 - 0.5; *p* = 0.52) was estimated for the change in BMI after diagnosis.

Penile cancer cases were significantly more likely to be overweight or obese as compared to controls (Table 2). Based on conditional logistic regression, as compared to men with a normal weight (BMI <25), the risk of invasive penile cancer increased with increasing obesity, with an odds ratio of 2.64 (95 % CI 1.81-3.86; *p* = 0.0103) for overweight men and 3.24 (95 % CI 2.07-5.08; *p* = 0.0002) for obese men. When BMI was treated as a continuous variable, the risk of invasive penile cancer increased by an estimated 53 % (OR 1.53, 95 % CI 1.29-1.81, *p* < 0.0001 for every five-unit increase in BMI [Table 2]).

**Table 2:** BMI summary statistics for cases vs controls. For cases, the predicted date-of-diagnosis BMI was used

	Cases	Controls	OR (95 % CI)	*p*-value
Body Mass Index Categories	N (%)	N (%)		
<25	44 (16.5)	279 (35.0)	1.0	Ref
25–29.9 (overweight)	153 (57.5)	377 (47.2)	2.64 (1.81–3.86)	0.0103
≥30 (obese)	69 (26.0)	142 (17.8)	3.24 (2.07–5.08)	0.0002
	Mean (SD)	Mean (SD)		
Body Mass Index (kg/m^2^)	28.4 (3.6)	26.8 (4.5)	1.53 (1.29–1.81)*	<0.0001

*Estimated effect for a 5-unit increase in BMI as a continuous variable

A sensitivity analysis was performed to assess the effect of using regression-derived estimates of BMI at diagnosis. This was accomplished by repeating the risk analysis using observed BMIs from the subset of cases who had DL issue dates prior to and within 5 years of diagnosis. Estimated odds ratios were higher in the sensitivity analysis, albeit with wider confidence intervals; thus suggesting that risk estimates are not artificially inflated by the use of case-estimated BMIs.

## Discussion

In this population-based case–control study, we found increasing BMI was associated with higher risk of developing invasive penile cancer. These results are consistent with our previous hospital-based study, which showed the odds of having invasive penile cancer doubled with each five-unit increase in BMI [3]. To our knowledge, this is the first population-based case–control study investigating the role of BMI in penile cancer incidence. Strengths of this study include the ability to capture a large number of penile cancer cases given its rarity and utilizing two large population-based databases (SHRI and the Iowa DLD) which are not subject to referral bias.

The link between obesity and cancer is gaining acceptance and it was recently reported that 3.6 % of all new cancers in 2012 were attributable to high BMI [6]. We hypothesize the association as it relates to penile cancer is secondary to obesity-related mechanisms such as impaired genital hygiene and self-examination, buried penis with smegma accumulation and functional phimosis; all risk factors for development of penile cancer. Obesity may also lead to diabetes, which increases the risk of phimosis and the risk of penile cancer [7]. Other, more systemic obesity-associated carcinogenesis mechanisms, such as chronic inflammation, oxidative stress and insulin resistance may also play a role in the development of penile cancer [8].

This study is limited by the inability to control for other risk factors of penile cancer such as circumcision status, smoking history and race. As circumcision is not associated with obesity, and 93 % of Iowa residents are Caucasian [9], these factors are unlikely to confound these results. Also, smoking is inversely associated with obesity [10], and any confounding due to smoking will bias the study towards the null and unlikely to explain our findings. Furthermore, in our previous case series these factors were controlled for and an association was still found [3]. A second limitation is the use of self-reported height and weight. This has been reported to underestimate obesity prevalence’s in men [11]; however, this underreporting should be consistent between cases and controls and will bias the study towards the null, minimizing the difference seen. Therefore, our results could actually underestimate the effects of obesity on penile cancer risk. Furthermore, the use of the Iowa DLD as a population-based sampling frame has been shown to include 97 % of men on the state’s tumor registry, therefore not excluding the less affluent who may be at highest risk of penile cancer [12]. Finally, temporal differences in BMI and the time of diagnosis necessitated the use of case-estimated BMIs; however, a sensitivity analysis indicated that this did not artificially inflate risk estimates.

## Conclusions

Many adverse health consequences result from obesity, including increased risk for several cancers. This study shows that obesity is associated with an increased risk of developing invasive penile cancer. Penile cancer is a morbid condition and therapeutic options for advanced cancer are limited. Obesity may represent a modifiable risk factor for the development of penile cancer. Greater emphasis on education of obese men about this risk might encourage weight loss and persuade them to perform periodic genital self-examination and seek care early.

## Abbreviations

BMI, body mass index; DLD, drivers’ license database; HPV, human papilloma virus; SEER, surveillance, epidemiology, and end results; SHRI, State Health Registry of Iowa; WHO, World Health Organization

## References

[1] Siegal R, Ma J, Zou Z, Jemal A (2014). Cancer statistics, 2014. CA Cancer J Clin.

[2] Daling J, Madeleine M, Johnson L (2005). Penile cancer: importance of circumcision, HPV and smoking in in situ and invasive disease. Int J Cancer.

[3] Barnes KT, Smith BJ, Lynch CF, Gupta A (2013). Obesity and invasive penile cancer. Eur Urol.

[4] State Health Registry of Iowa. Iowa Cancer Registry. http://www.public-health.uiowa.edu/shri/. Accessed 9 October 2014.

[5] World Health Organization. Global Database on Body Mass Index. BMI Classification. http://apps.who.int/bmi/index.jsp?introPage=intro_3.html. Accessed 9 October 2014.

[6] Arnold M, Pandeya N, Byrnes G (2015). Global burden of cancer attributable to high body-mass index in 2012: a population-based study. Lancet Oncol.

[7] Bromage S, Crump A, Pearce I (2008). Phimosis as a presenting feature of diabetes. BJU Int.

[8] Basen-Engquist K, Chang M (2011). Obesity and cancer risk: recent review and evidence. Curr Oncol Rep.

[9] U.S. Census Bureau. State and County Quick Facts. http://quickfacts.census.gov/qfd/states/19000.html. Accessed 1 November 2014.

[10] Flegal K, Troiano R, Pamuik E, Kuczmarski RJ, Campmbell SM (1995). The influence of smoking cessation on the prevalence of overweight in the United States. NEJM.

[11] Morris D, Schubert S, Ngo D, Moore JM. DMV records are valuable for monitoring obesity in Oregon. Oregon Health Authority Environmental Public Health Tracking. http://public.health.oregon.gov/HealthyEnvironments/TrackingAssessment/EnvironmentalPublicHealthTracking/Documents/Reports/EPHT_DMV_obesity_tracking.pdf. Accessed 13 December 2014.

[12] Lynch C, Logsden-Sackett N, Edwards S, Cantor KP (1994). The driver’s license list as a population-based sampling frame in Iowa. Am J Public Health.

